# Integrative Analyses and Verification of the Expression and Prognostic Significance for RCN1 in Glioblastoma Multiforme

**DOI:** 10.3389/fmolb.2021.736947

**Published:** 2021-10-13

**Authors:** Weicheng Lu, Hong Chen, Bo Liang, Chaopeng Ou, Mingwei Zhang, Qiuyuan Yue, Jingdun Xie

**Affiliations:** ^1^ State Key Laboratory of Oncology in Southern China, Department of Anesthesiology, Sun Yat-sen University Cancer Center, Collaborative Innovation for Cancer Medicine, Guangzhou, China; ^2^ Department of Gastrointestinal Surgery, Fujian Provincial Hospital, Fuzhou, China; ^3^ Nanjing University of Chinese Medicine, Nanjing, China; ^4^ Department of Radiation Oncology, The First Affiliated Hospital of Fujian Medical University, Fuzhou, China; ^5^ Department of Radiology, Fujian Cancer Hospital and Fujian Medical University Cancer Hospital, Fuzhou, China

**Keywords:** glioblastoma multiform, invasion, prognosis, nomogram, RCN1

## Abstract

Glioblastoma multiform is a lethal primary brain tumor derived from astrocytic, with a poor prognosis in adults. Reticulocalbin-1 (RCN1) is a calcium-binding protein, dysregulation of which contributes to tumorigenesis and progression in various cancers. The present study aimed to identify the impact of RCN1 on the outcomes of patients with Glioblastoma multiforme (GBM). The study applied two public databases to require RNA sequencing data of Glioblastoma multiform samples with clinical data for the construction of a training set and a validation set, respectively. We used bioinformatic analyses to determine that RCN1 could be an independent factor for the overall survival of Glioblastoma multiform patients. In the training set, the study constructed a predictive prognostic model based on the combination of RCN1 with various clinical parameters for overall survival at 0.5-, 1.0-, and 1.5-years, as well as developed a nomogram, which was further validated by validation set. Pathways analyses indicated that RCN1 was involved in KEAS and MYC pathways and apoptosis. *In vitro* experiments indicated that RCN1 promoted cell invasion of Glioblastoma multiform cells. These results illustrated the prognostic role of RCN1 for overall survival in Glioblastoma multiform patients, indicated the promotion of RCN1 in cell invasion, and suggested the probability of RCN1 as a potential targeted molecule for treatment in Glioblastoma multiform.

## Introduction

Glioblastoma multiforme (GBM) represents the most prevalent brain cancer in adults and has a dismal prognosis and poor quality of life (Omuro and DeAngelis, 2013). The current treatment strategies for GBM are maximum surgical resection followed by a combination of chemotherapy and radiotherapy (Gilbert et al., 2013; Ostrom et al., 2015). Even with the advancement in therapeutic options over recent decades, recent studies have demonstrated that the median survival of GBM patients is 16.6 months, which decreases after 2 years, with a survival rate of only 34% (Gilbert et al., 2013). Several studies have illustrated that some omics markers within tumors could impact patients’ survival, like the status of Isocitrate dehydrogenase 1/2 (IDH1/2) mutation, glioma-CpG island methylator phenotype (G-CIMP), methylation of O-6-methylguanine-DNA methyltransferase (MGMT), and codeletion for chromosome 1p and 19q (1p/19q codeletion) (Hartmann et al., 2010; Wick et al., 2013; Hainfellner et al., 2014; Louis et al., 2016). In addition, further improvements have been made with subspecialized care, improved resection methods precisely targeted radiotherapy, and early systemic salvage therapies (Jayamanne et al., 2018). However, patients with GBM still have a poor prognosis due to the GBM’s aggressive behavior, rapid progression, and frequent recurrence (Soffietti et al., 2014). Thus, it is imperative to search for a novel biomarker with good prediction for the prognostic signature of GBM via various methods, to explore the molecular mechanisms for precisely targeted treatments in GBM.

Reticulocalbin-1 (RCN1), a calcium-binding protein, contains six conserved regions and is located in the endoplasmic reticulum (Ozawa and Muramatsu, 1993), which regulates calcium-dependent activities combined with reticulocalbin 2 (RCN2) (Nakakido et al., 2016). Dysregulation of RCN1 protein has been reported in multifarious diseases, including cancer, cardiovascular, and neuromuscular diseases (Grzeskowiak et al., 2003; Liu et al., 1997; Zhang et al., 2006). It has been found that RCN1 is involved in breast cancer (Nakakido et al., 2016), colorectal cancer (Nimmrich et al., 2000), liver cancer (Lu et al., 2015), kidney cancer (Giribaldi et al., 2013), and non-small cell lung cancer (Chen et al., 2019). In addition, it is reported that down-regulation of RCN1 facilitates apoptosis and necroptosis in prostate cancer cells (Liu et al., 2018). Overall, the above findings have revealed that dysregulation of RCN1 could contribute to tumorigenesis and progression. However, the relationship between RCN1 and prognosis, nor its biological functions in GBM, have been completely investigated.

**FIGURE 1:**
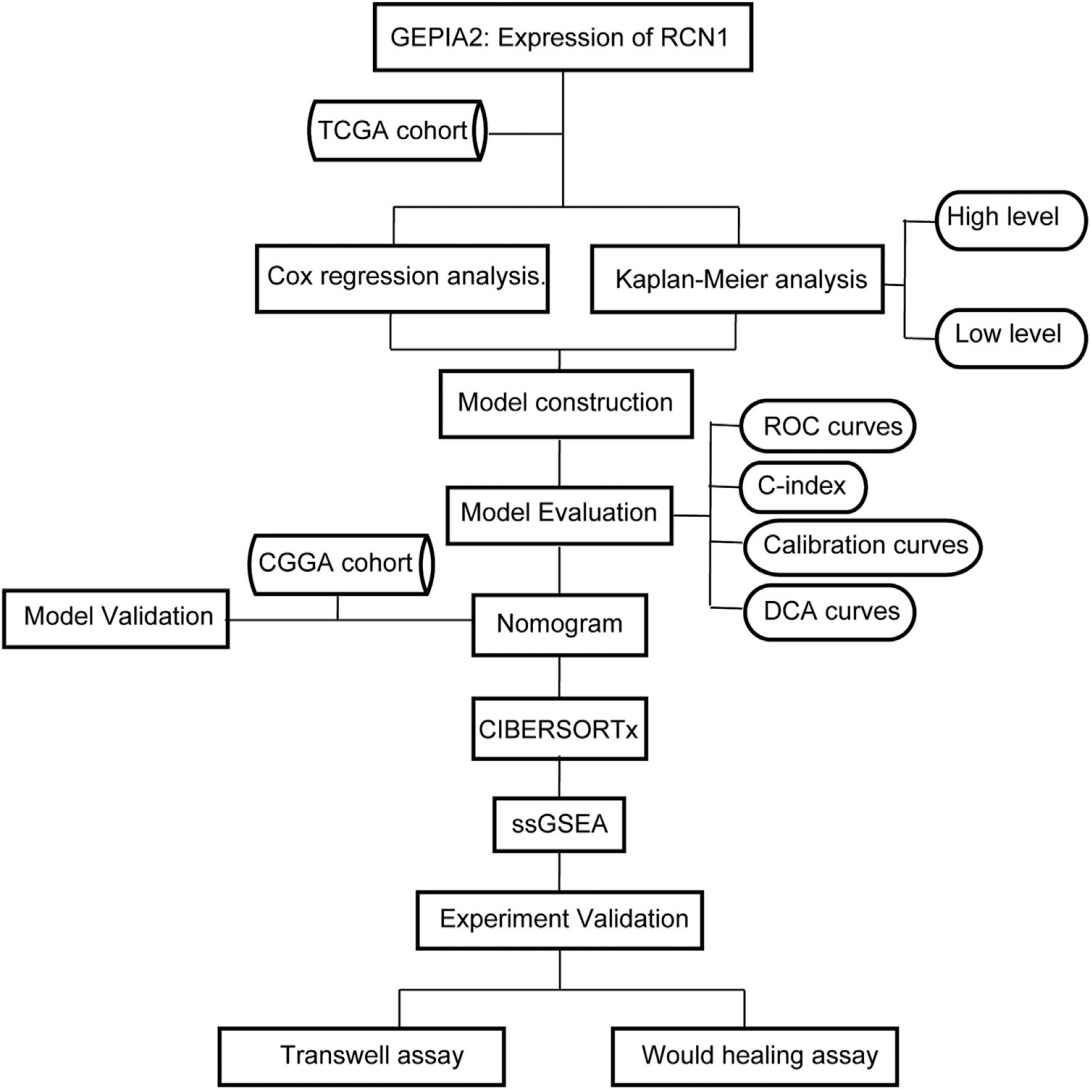
Flow chart of this study.

In our study, GBM samples in the Cancer Genome Atlas (TCGA) database were enrolled in the training set, and cases in the Chinese Glioma Genome Atlas (CGGA) were used for the external validation set, to assess the RCN1-related prognostic signature in GBM. Gene Expression Profiling and Interactive Analyses (GEPIA2) (Tang et al., 2017) (http://gepia2.cancer-pku.cn/#index) were used to profile the tissue-wise expression of RCN1 in GBM. In addition, the next version of Cell type Identification By Estimating Relative Subsets Of RNA Transcripts (CIBERSORTx, (Newman et al., 2019) https://cibersortx.stanford.edu/) was used to illustrate the abundances of the infiltration immune cells correlating with expression of RCN1 in GBM, and the single sample gene search enrichment analyses (ssGSEA) (Barbie et al., 2009) was utilized to determine potential pathways of RCN1 involved (Figure 1).

## Materials and Methods

### Data Acquisition

The RNA sequencing data (level 3) of GBM with corresponding clinical data were downloaded from the TCGA database to assess the prognostic impact of RCN1-signature on patients with GBM, and were enrolled into the training set. Then, GBM samples from the Chinese Glioma Genome Atlas (CGGA) dataset were employed as an external validation set for further verification.

### Differential Analysis of Reticulocalbin-1 Expression in Glioblastoma Multiforme

Gene Expression Profiling and Interactive Analyses (GEPIA) (Tang et al., 2017) is an online resource for gene expression analysis, and GEPIA2 (http://gepia2.cancer-pku.cn/#index) (Tang et al., 2019) was an updated version of GEPIA, containing 163 GBM cases of TCGA and 207 normal brain samples of the Genome Tissues Expression database (GETx) (GTEx Consortium, 2015). We performed a differential analysis of RCN1 expression through GEPIA2 and used a boxplot to show the results with log2 of transcript count per million [log_2_ (TPM + 1)] presenting the expression level of RCN1.

### Survival Analysis

As described previously (Ogłuszka et al., 2019), we used *survMisc* package in R to divide GBM samples into high and low RCN1 groups based on the optimum cutoff. For the exploration of the association between the expression of RCN1 and survival, Kaplan-Meier (KM) analysis with log-rank test was conducted by using *survival* package.

### Evaluation of Reticulocalbin-1-Based Prognostic Value

To explore the effect of the variation of RCN1 expression on OS in GBM patients, we estimated the relative risk and log2-based transformation for survival status, followed by fitting smooth line using a locally estimated scatterplot smoothing (LOESS) method (Zheng et al., 2020). We then compared the predictive effect of RCN1 with different prognostic factors by receiver operating characteristic (ROC) curve (Zhou et al., 2019). We regarded the RCN1 expression as a single continuous covariate for further regression analysis based on the result of LOESS analysis. We adjusted for clinical features, which changed the regression coefficients of RCN1 by more than 10%, or of *p* < 0.1 through the univariate analysis (Kernan et al., 2000). Next, adjust I model was determined after adjusting confounders, while all clinical factors were enrolled as adjust II model. Then, univariate and multivariate analyses using Cox proportional hazard regression model were performed. We also applied LOESS method to visually assess the relationship between RCN1 expression and OS in adjust I and adjust II models, respectively. Interaction test and stratified analysis (Soria et al., 2015) were also carried out to assess the differential prognostic value of RCN1 in accordance with a different model. A two-tailed *p* < 0.05 was considered to be statistically significant.

### Construction and Comparison of Three Prognostic Models

After determining the prognostic features, five clinical prognostic factors in GBM (age, gender, IDH status, chemotherapy, as well as radiotherapy) were enrolled to construct the prognostic model as model 1. Model 2 (the expression of RCN1) and model 3 (model 1 + model 2) were compared with model 1 to estimate the robustness of different prognostic features to GBM prognosis, respectively. We used discrimination, calibration, and model improvement capability to evaluate the different models. The discrimination of these models was estimated by ROC curve, concordance index (C-index) (Harrell et al., 1996), as well as decision curve analysis (DCA) (Vickers and Elkin, 2006; Vickers et al., 2008; Rousson and Zumbrunn, 2011; Kerr et al., 2016). Three corresponding models were constructed with *coxph* function in *survival* package, and then the risk value of patients in each model was calculated with *predict* function, and thus the ROC curves were constructed by *timeROC* (Heagerty et al., 2000; Blanche et al., 2013) with those risk values. Three models were firstly built with *cph* function, and then their C-index at different times with the prediction error curve was calculated by *cindex* function in *pec* package. The *stdca* package was applied to visualization for DCA. The calibration curves of different models were completed via *caoplot* function in *pec* package. Notably, we used the bootstrap method with 1,000 resamples both for analyses of discrimination and calibration. Moreover, the improvement capability of the model was assessed through the net reclassification improvement (NRI) and the integrated discrimination improvement (IDI) by using *IDI. INF.OUT* function in *survIDINRI* package (Pencina et al., 2008). After the best model was determined, *regplot* package was employed to construct the diagram of the nomogram.

### External Validation of the Prognostic Signature

The prognostic capability of the RCN1-based signature was externally validated in the validation set. The gene expression information with corresponding clinical factors of GBM was obtained from the CGGA database. Similarly, we divided GBM patients into high- and low-RCN1 groups by the optimum cut-off value, and then performed the KM survival curve. Afterward, using *predict* function, the time-independent ROC curves, C-index, and calibration curves were employed to assess the accuracy of prognostic model RCN1 signature-based.

### Infiltrative Immune Cell Analysis

CIBERSORTx (Newman et al., 2019), the next-generation version of CIBERSORT, a machine learning tool, provides an estimation of the abundances of member cell types in a condition with a mixed cell population, by using gene expression data (Newman et al., 2019). We applied CIBERSORTx to analyze the infiltration level of 22 immune cells in high- and low-RCN1 groups, using samples from TCGA and CGGA datasets, respectively. After enabling batch correction, performing the “Bulk mode”, and selecting the quantile normalization algorithm, the results were represented with an absolute score for the proportion of 22 immune cell subsets of GBM samples. Consecutively, the samples with *p* < 0.05 were retained after repeating the crossover operation 500 times (Ali et al., 2016). Wilcoxon rank-sum test was applied to identify the differences between the two groups.

### ssGSEA

The ssGSEA was used to identify the differentially enriched hallmarks for a single sample (Barbie et al., 2009). To identify key pathways related to RCN1, we chose to focus on 50 hallmark gene sets, which were designed to highlight gene sets contained in the Molecular Signatures Database (MSigDB) (Subramanian et al., 2005). Gene symbol profiles for *homo sapiens* were downloaded from MSigDB database (Liberzon et al., 2015). Then we estimated the degree of each hallmark’s ssGSEA profile in two groups, using the *gsva* package, both in the training and validation cohort. Next, by *limma* package, differential analysis was performed; and |*t*| > 1 or adjusted *p* < 0.05 were considered as statistically significant.

### Validation of the Effect of Reticulocalbin-1 on Glioblastoma Multiforme Cells

#### Cell Culture

Human GBM cell lines U87 and A172 were purchased from American Type Culture Collection (ATCC, Manassas, Virginia, United States), which were authenticated with a short tandem repeat. Cells were set on a humidified incubator with 5% CO_2_ at 37°C, as well as cultured in Dulbecco’s Modified Eagle’s Medium (GIBCO, Billings, MT, United States) added with 10% fetal bovine serum (GIBCO).

#### Small Interfering RNA Transfection

The small interfering RNA of RCN1 (si-RCN1) sequences and the corresponding negative control were designed and purchased from RiboBio (Guangzhou, China). For transient silencing, A172 and U87 GBM cell lines were transfected with negative control or si-RCN1 by Lipofectamine^TM^ 3,000 Reagent (Invitrogen, United States) according to the manufacturer’s instruction. After 48 h, cells were harvested for quantitative real-time-polymerase chain reaction (qRT-PCR) analysis. Target sequences for transient silencing were as follows: si-RCN1-1: GAA​GCT​AAC​TAA​AGA​GGA​A; si-RCN1-2: CCA​GGC​ATC​TGG​TAT​ATG​A; negative control siRNA was obtained from RiboBio (Guangzhou, China).

#### qRT-PCR

By using ReverTra Ace® qPCR RT Master Mix with gDNA Remover (TOYOBO, Shanghai, China), total RNA was extracted and then was used to synthesize the first complementary DNA (cDNA) strand according to the manufacturer’s protocol. The qRT-PCR reaction was carried out to estimate the RNA levels and *ACTIN* was used as the internal reference. The primers used for qRT-PCR were as follows: RCN1 forward 5′-AAG​GAG​AGG​CTA​GGG​AAG​ATT-3′ and reverse 5′-ATC​CAG​GTT​TTC​AGC​TCC​TCA-3’; ACTIN forward 5′- CAC​CAT​TGG​CAA​TGA​GCG​GTT​C-3′ and reverse 5′- AGG​TCT​TTG​CGG​ATG​TCC​ACG​T -3’. The relative normalized expression of the target genes was compared with that of ACTIN, and the mRNA expression of each gene was calculated with the 2^−ΔΔCt^ method (Liang et al., 2021).

#### Cell Invasion Assays

Cell invasion was measured through wound healing and transwell migration assays following the manufacturers’ instructions. In brief, cells were plated in 6-well plates and cultured at 37°C with 5% CO_2_. After 24 h, at which cells were reached on 80% confluence, we used a sterile 10 μL disposable serological pipette to make a straight-line scratch, and then cells were harvested after 48 h. Images of the scratch width were taken using an inverted microscope (Olympus IX73 Inverted Microscope, Olympus, Beijing China) at 0 and 48 h after the scratch, and then calculated by ImageJ software (version 1.52, National Institutes of Health, United States).

As for the transwell migration assay, it was performed using the Boyden chamber with a gelatin-coated polycarbonate filter with an 8-μm pore size (Neuro Probe, Gaithersburg, MD, United States). Cells were added to the upper chamber in 24-well plates at a density of 5.0 × 10^4^ cells per well, and the lower chamber was filled with 800 μL 10% FBS for 24 h. Cells in the transwell chamber were fixed with 4% paraformaldehyde for 15 min, stained with 0.1% crystal violet for 30 min, and then observed by a BDS500 Inverted Biological Microscope (Chongqing Optec Instrument Co., Ltd., China).

#### Statistical Analysis

All the data were presented as mean ± standard deviation and the statistical analyses were performed by IBM SPSS Statistics 25.0 software (International Business Machines Corporation, United States). The student’s t-test was performed to evaluate the significant difference between the two groups.

## Results

### Patients Characteristics

A total of 361 GBM samples (153 patients from TCGA as the training cohort and 208 patients from CGGA as the validation cohort) were obtained in our study, as shown in Table 1.

**TABLE 1:** The clinical characteristics of patients and expression of RCN1 in GBM.

Characteristics	TCGA (*N* = 153)	CGGA (*N* = 208)
Age	60 (52∼69)	53 (43∼60)
Male	54 (35.29%)	82 (39.42%)
RCN1	59.60 ± 12.29	6.12 (5.14∼6.62)
IDH status		
Mutant	10 (6.54%)	31 (14.90%)
Wild type	143 (93.46%)	177 (85.10%)
Radiotherapy		
Yes	130 (84.97%)	184 (88.46%)
No	23 (15.03%)	24 (11.54%)
Chemotherapy		
Yes	112 (73.20%)	168 (80.77%)
No	41 (26.80%)	40 (19.23%)

Values are expressed as median (interquartile range), number of cases (%), or mean ± standard deviation. TCGA: The Cancer Genome Atlas, CGGA: Chinese Glioma Genome Atlas, RCN1: Reticulocalbin-1, IDH: isocitrate dehydrogenase.

### Reticulocalbin-1 Was Elevated in Glioblastoma Multiforme and May Act as an Oncogene

**FIGURE 2:**
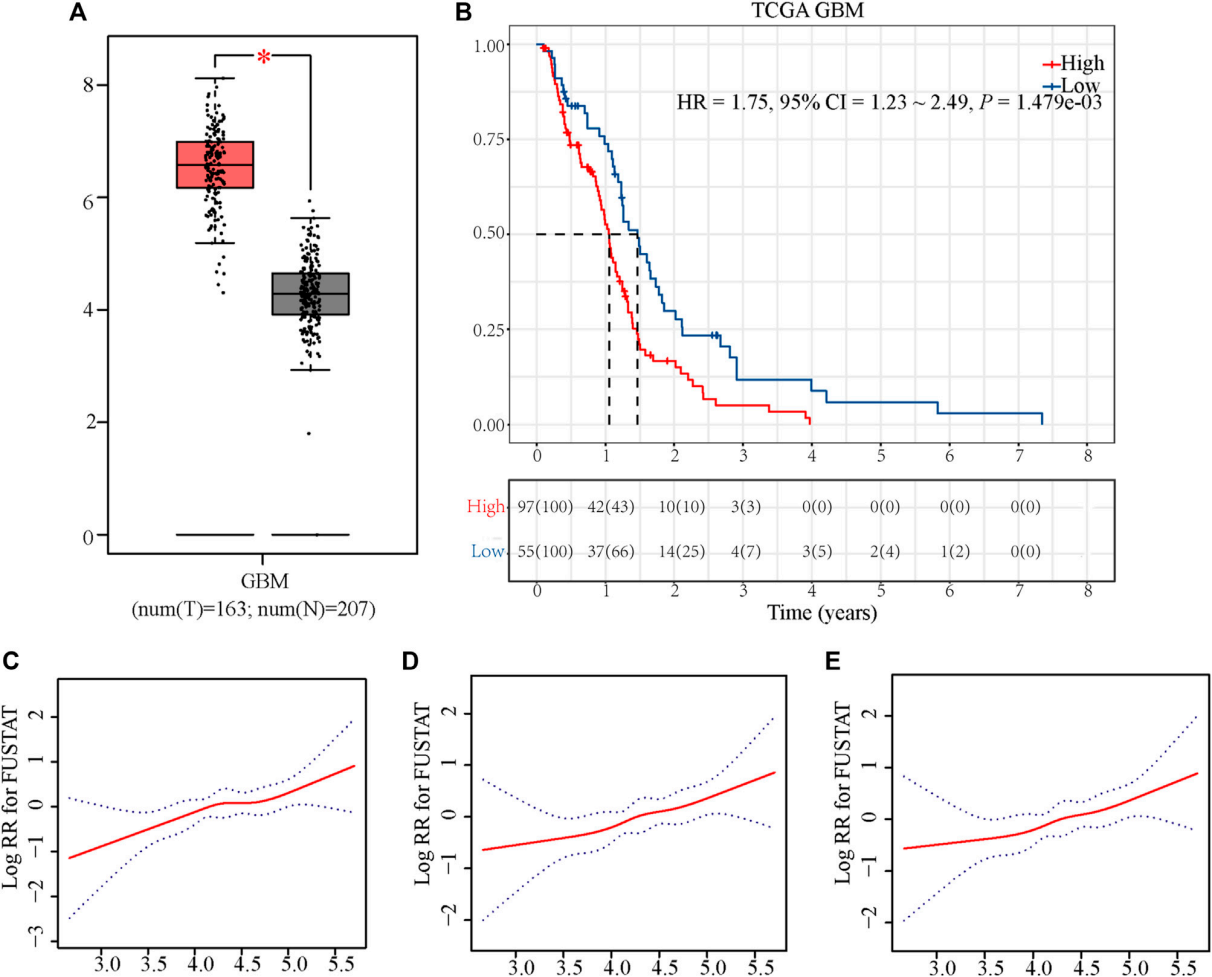
Exploration of the association between RCN1 and OS of patients with GBM in TCGA. **(A)** Boxplot. **(B)** Kaplan-Meier curve with log-rank test in TCGA. **(C)** Association between the variation of RCN1 expression and OS in non-adjusted. **(D)** Association between the variation of RCN1 expression and OS in adjusted I model. **(E)** Association between the variation of RCN1 expression and OS in adjusted II model.

As shown in Figure 2A, we found that the expression of RCN1 was higher in the GBM samples (*n* = 163) than in the normal brain tissues (*n* = 207), by using the GEPIA2 tool. In the TCGA database, as the near-linear correlation between the variation of RCN1 expression and OS revealed through LOESS (Figure 2C), RCN1 expression was considered as a single continuous variation for further analysis. A total of 153 samples were clustered into the high- (*n* = 97) or low-RCN1 group (*n* = 56) by the optimal cut-off value (4.144). Patients with higher RCN1 expression had a worse OS than those with a low one in GBM (*p* = 0.001) (Figure 2B).

### Reticulocalbin-1 is an Independent Prognostic Signature for Glioblastoma Multiforme

**FIGURE 3:**
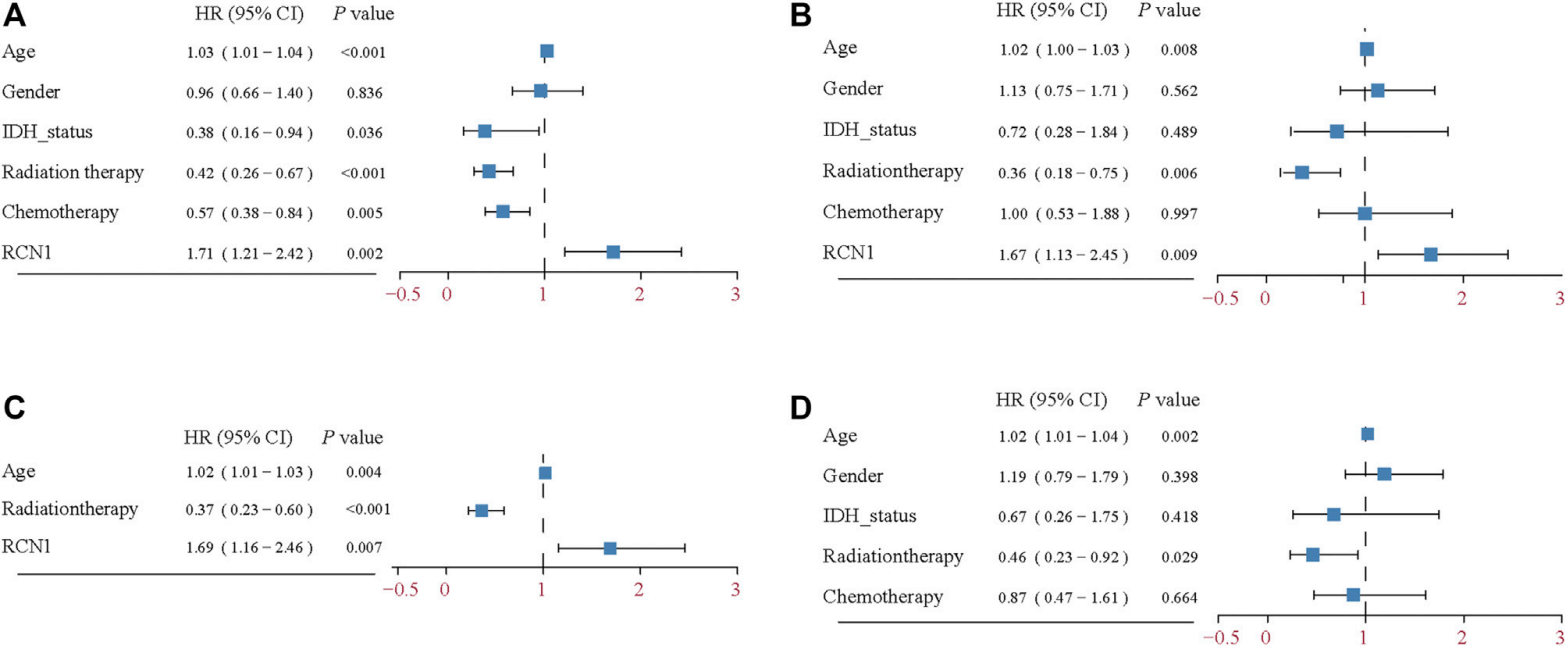
Forest plot of the Cox regression analysis. **(A)** Univariable Cox regression analysis for the training cohort. **(B)** Multivariable Cox regression analysis for the training cohort. **(C)** Multivariable Cox regression analysis for adjusted I model in the training cohort. **(D)** Multivariable Cox regression analysis for clinical features in training cohort.

Considering the interference of the confounding factors, identifying and then adjusting for potential confounding factors was conducted. We firstly found that RCN1 may be an independent prognostic signature when compared with other signatures (Supplementary Figure S1). In the training cohort, we then identified variants (age and radiotherapy) to be adjusted and then enrolled these clinical features into the adjusted I model. The adjustments for age, gender, IDH status, chemotherapy, and radiotherapy were included in the adjusted II model. Both non-adjusted and two adjusted models were analyzed by using the Cox regression analysis to further investigate whether RCN1 could estimate OS independently (Figure 3). As shown in Table 2, in the non-adjusted model, prognosis was correlated with age (HR = 1.03, 95% CI 1.01∼1.04, *p* < 0.001), radiotherapy (HR = 0.42, 95% CI: 0.26∼0.67, *p* < 0.001), IDH status (HR = 0.38, 95% CI: 0.16∼0.94, *p* = 0.036), chemotherapy (HR = 0.57, 95% CI 0.38∼0.84, *p* = 0.005), and RCN1 expression (HR = 1.71, 95% CI 1.21∼2.42, *p* = 0.002) in the training cohort. In adjust I model, after adjusting for confounding factors (age and radiotherapy), RCN1 was still associated with OS (HR = 1.69, 95% CI 1.16∼2.46, *p* = 0.007) (Table 2). Furthermore, after adjusting for five predominant clinical features (age, gender, IDH status, chemotherapy, and radiotherapy), RCN1 independently predicted prognosis in the training cohort (HR = 1.67, 95% CI 1.13∼2.45, *p* = 0.009) (Table 2). In the same way, we also found the near-linear correlation between the variation of RCN1 expression and OS both in adjust I model (Figure 2D) and adjust II model (Figure 2E), thus, we enrolled RCN1 as a single continuous variation for further analysis. In addition, subgroup analysis showed that there were no statistical differences neither in the non-adjusted model nor adjusted I model, except for chemotherapy (*P*_interaction_ = 0.0019 in the non-adjusted model, *P*_interaction_ = 0.0118 in the adjusted I model, respectively) (Table 3), revealing that RCN1 might be an independent prognostic factor for OS in patients with GBM.

**TABLE 2:** Cox proportional hazards regression models of various prognostic parameters in patients with GBM in TCGA.

Characteristic	Non-adjusted model		Adjusted I model		Adjusted II model	
	HR (95% CI)	*p* value	HR (95% CI)	*p* value	HR (95% CI)	*p* value
Age	1.03 (1.01–1.04)	<0.001	1.02 (1.01–1.03)	0.004	1.02 (1.0–1.03)	0.008
Gender	0.96 (0.66–1.4)	0.836	—	—	1.13 (0.75–1.71)	0.562
IDH status	0.38 (0.16–0.94)	0.036	—	—	0.72 (0.28–1.84)	0.489
Radiotherapy	0.42 (0.26–0.67)	<0.001	0.37 (0.23–0.6)	<0.001	0.36 (0.18–0.75)	0.006
Chemotherapy	0.57 (0.38–0.84)	0.005	—	—	1.0 (0.53–1.88)	0.997
RCN1	1.71 (1.21–2.42)	0.002	1.69 (1.16–2.46)	0.007	1.67 (1.13–2.45)	0.009

Adjusted I model: Factors including age, radiotherapy for adjustment, Adjusted II model: Factors including age, gender, IDH, radiotherapy and chemotherapy for adjustment. HR: hazard ratio, CI: confidence interval.

**TABLE 3:** Subgroup analysis of the associations between OS and RCN1 of patients with GBM in TCGA.

Covariates	Total (*N* = 153)	RCN1		RCN1^a^	
		HR (95% CI)	*P* for interaction	HR (95%CI)	*P* for interaction
Age	—	—	0.2553	—	—
<60 y	72	2.02 (1.24–3.29)^c^	—	—	—
>=60 y	81	1.36 (0.84–2.21)	—	—	—
Total	153	1.66 (1.16–2.36)^c^	—	—	—
Gender	—	—	0.8929	—	0.7652
Male	54	1.67 (0.98–2.83)	—	1.83 (0.96–3.51)	—
Female	99	1.75 (1.11–2.75)^b^	—	1.62 (1.00–2.63)	—
Total	153	1.71 (1.21–2.42)^c^	—	1.69 (1.15–2.50)^c^	—
Radiotherapy	—	—	0.6713	—	—
No	23	2.70 (0.50–14.62)	—	—	—
Yes	130	1.86 (1.28–2.70)^c^	—	—	—
Total	153	1.89 (1.31–2.73)^d^	—	—	—
Chemotherapy	—	—	0.0019	—	0.0118
No	41	0.62 (0.29–1.33)	—	0.81 (0.38–1.73)	—
Yes	112	2.33 (1.53–3.53)^d^	—	2.55 (1.63–3.98)^d^	—
Total	153	1.66 (1.17–2.34)^c^	—	1.91 (1.30–2.80)^c^	—

a Covariates were adjusted as in Adjusted I model (Table 2). HR (95% CI) were derived from Cox proportional hazards regression models.

b *P* < 0.05.

c < 0.01.

d *P* < 0.001. HR: hazard ratio, CI: confidence interval.

### Construction and Evaluation of Three Prognostic Models

**FIGURE 4:**
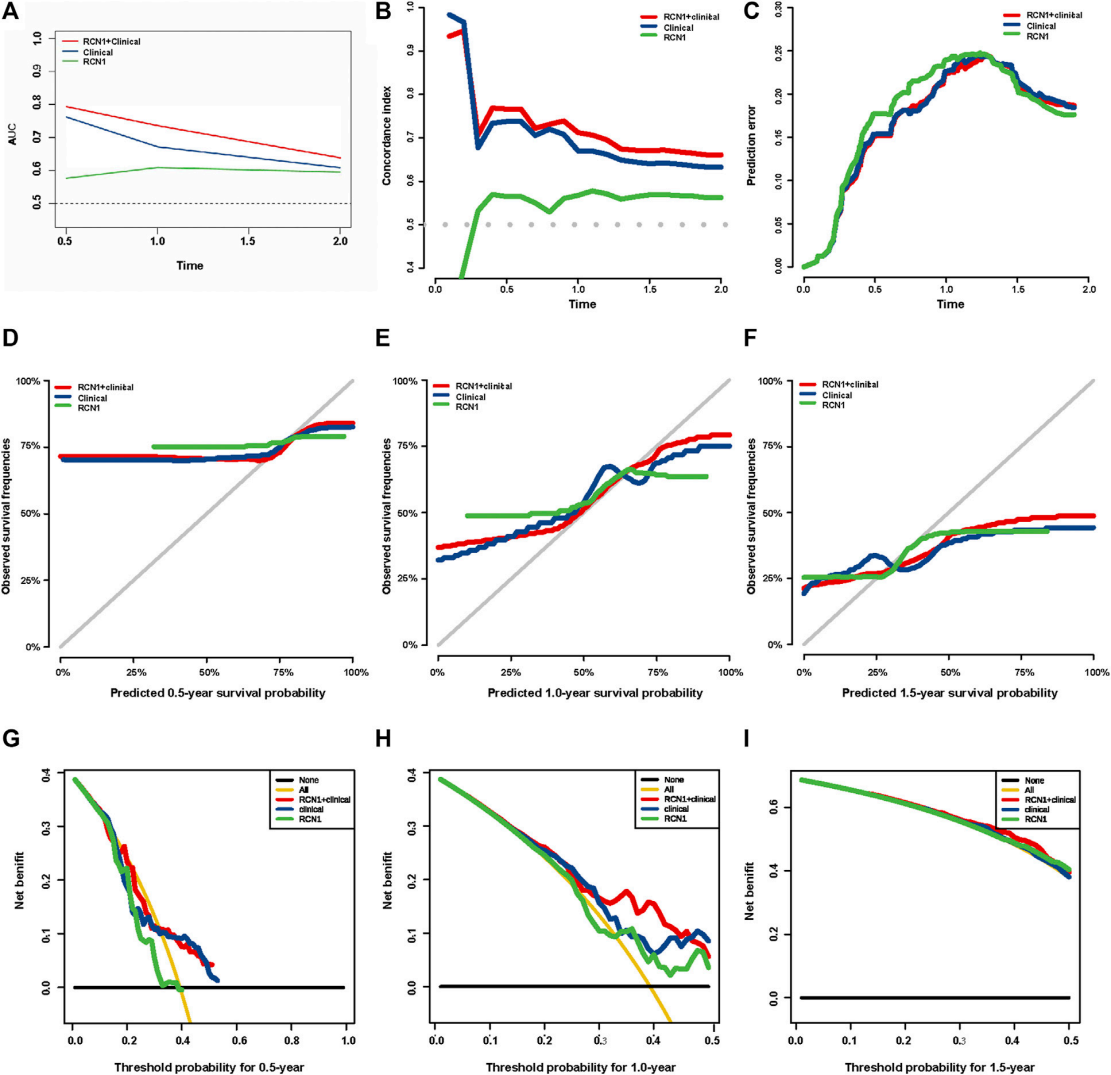
Evaluation of three prognostic models in TCGA. **(A)** ROC curves of the three models at different times. **(B)** C-index curves of the three models at different times. **(C)** Prediction error probability curves of the three models at different times. **(D–F)** Calibration curves of the three models at different times. **(G–I)** DCA curves of the three models at different times.

**FIGURE 5:**
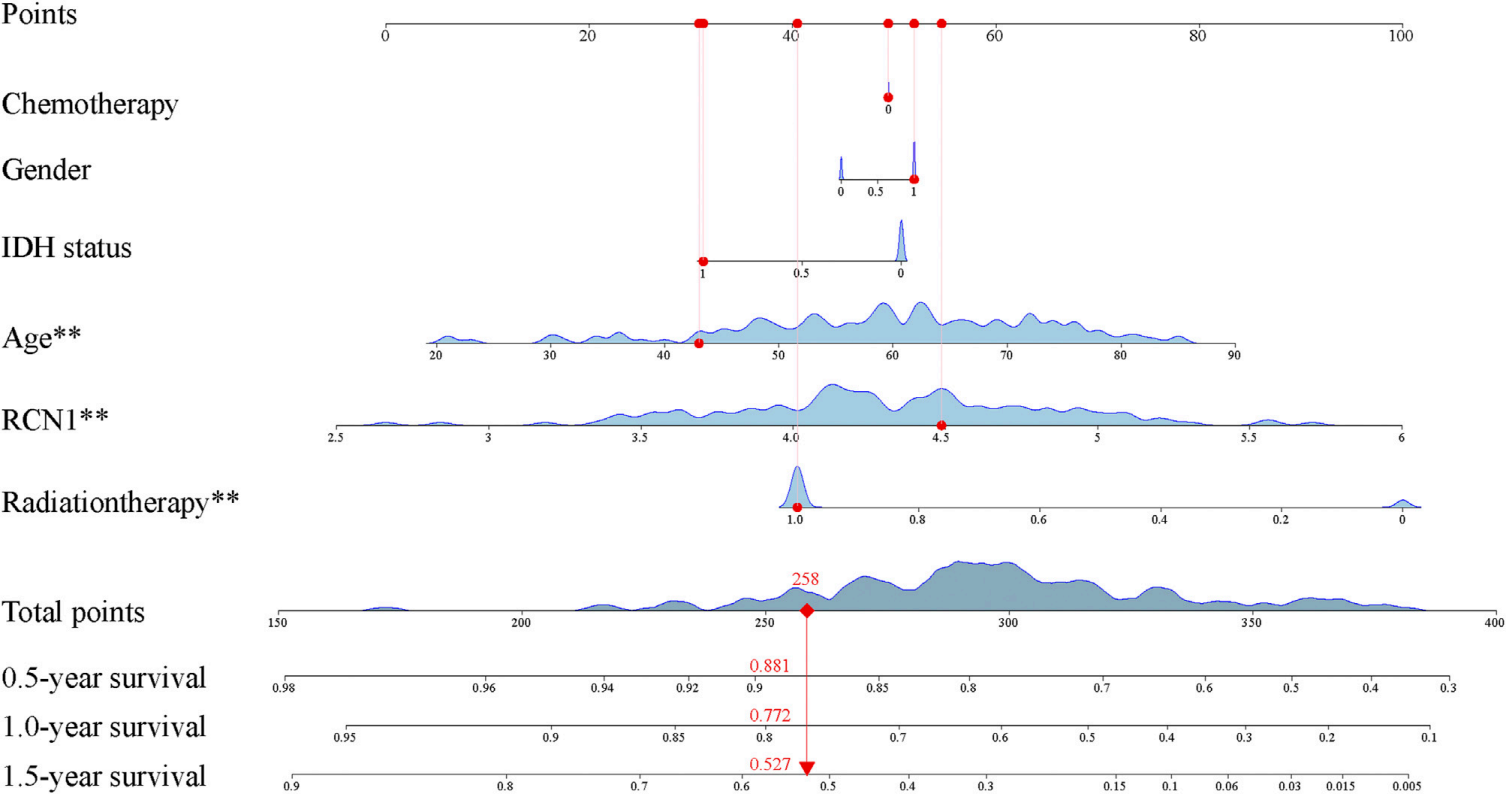
Construction of prognostic nomogram.

The clinical features and RCN1 were enrolled to construct the prognostic models for GBM. We firstly built three prognostic models (model 1: five clinical variants, model 2: the expression of RCN1, and model 3: model 1 + model 2) and then evaluated them. Model 3 had a higher area of under curve (AUC), better C-index, and lower prediction error compared with model 2 and model 1 (Figures 4A–C). DCA showed that the net benefit of model 3 in 0.5 and 1 year is better than the other two models, but there was no significant difference in 1.5 years (Figures 4D–F). It was found that the calibration of model 3 was better than that of model 1 and model 2 in 0.5 and 1 year, while the calibration of the three models was poor in 1.5 years (model 2 was better than others) (Figures 4G–I). As for model improvement capability, when model 1 was considered as the reference, the NRI and IDI of model 3 were both positive, in which the NRI of 1.5 years was increased by 16.7% (*p* = 0.064) and the IDI of 1.5 years was increased by 3.7% (*p* = 0.034); on the contrary, the NRI and IDI of model 2 were both negative, where the IDI of 0.5 year was decreased by 11.4% (*p* = 0.0016) meanwhile the NRI was decreased by 27.4% (*p* = 0.040) (Table 4). From the above results, it can be determined that model 3 had good discrimination and calibration in the prediction of OS. Therefore, we developed a nomogram in accordance with model 3 to assess OS at 0.5-, 1.0-, and 1.5-years in the TCGA dataset, in which each signature was assigned points according to its risk contribution to OS (Figure 5).

**TABLE 4:** The model improvement capability in three prognostic models.

Year	Model 3 vs Model 1				Model 2 vs Model 1			
	NRI		IDI		NRI		IDI	
	Value	*p* value	Value	*p* value	Value	*p* value	Value	*p* value
0.5	0.129	0.284	0.018	0.176	−0.274	0.040	−0.114	0.016
1.0	0.122	0.156	0.024	0.094	−0.082	0.384	−0.088	0.056
1.5	0.164	0.064	0.037	0.034	−0.035	0.707	−0.031	0.322

Model 1 represents the prognostic model with age, gender, IDH status, chemotherapy, and radiotherapy; Model 2 only comprises the expression of RCN1; Model 3 integration of model 1 and 2, including all factors (age, gender, IDH status, chemotherapy, radiotherapy, and the expression of RCN.

### External Validation for Nomogram in Chinese Glioma Genome Atlas

**FIGURE 6:**
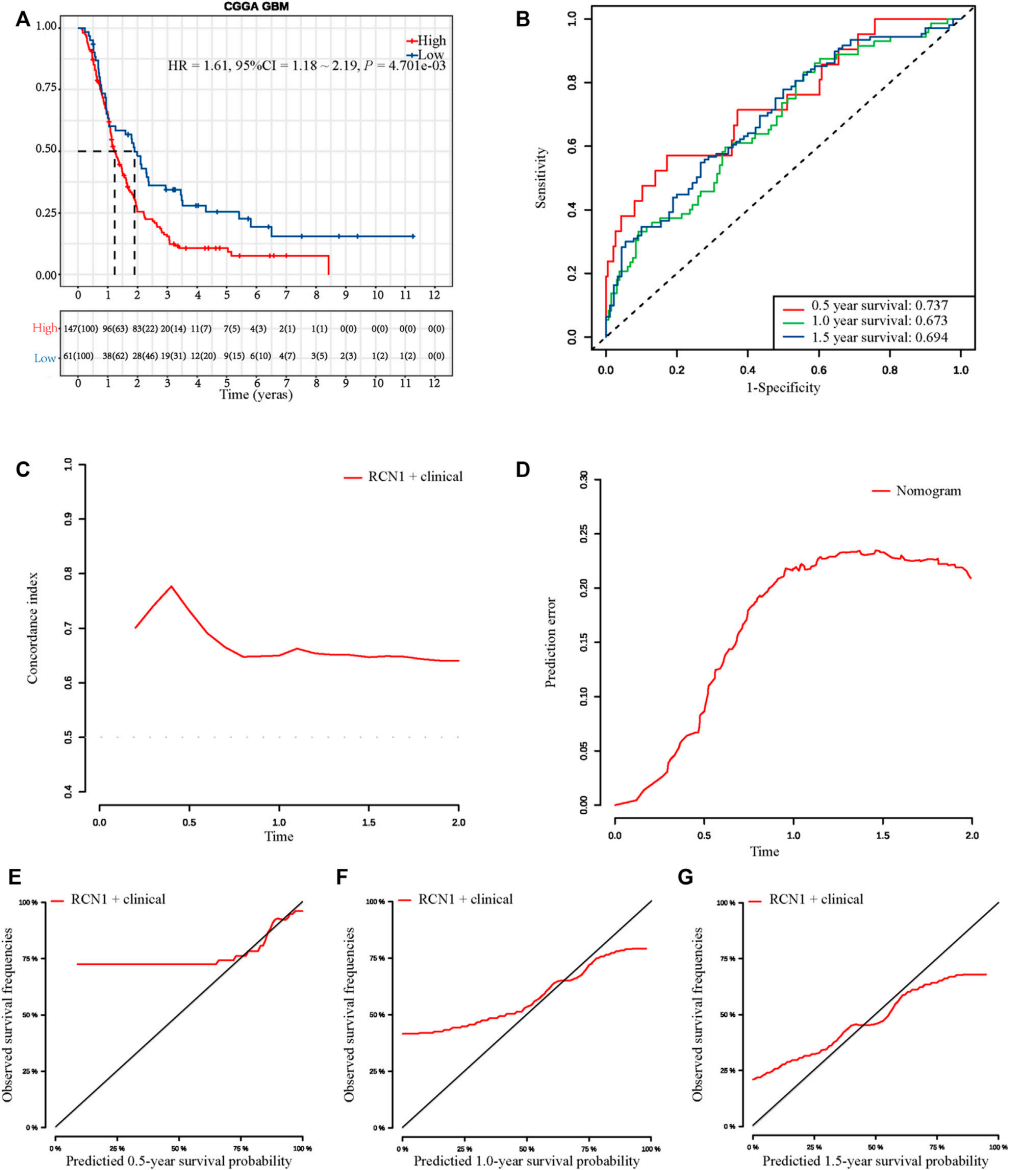
External validation of the prognostic signature in CGGA. **(A)** Kaplan-Meier curve with log-rank test in CGGA. **(B)** ROC curves of the three models at different times. **(C)** C-index curves of the three models at different times. **(D)** Prediction error probability curves of the three models at different times. **(E–G)** Calibration curves of the three models at different times.

To assess whether an RCN1 signature-based model can similarly play a prognostic value in different populations, a total of 208 GBM samples from the CGGA database as an external validation cohort were used to assess its prediction performance. According to the optimum cutoff (5.521), high- (*n* = 147) and low- (*n* = 61) RCN1 groups were determined. Consistent with the findings in the training cohort, the Kaplan-Meier curve revealed patients with high RCN1 represented a worse OS than those with low expression (*p* = 0.0047) (Figure 6A). Moreover, the time-dependent ROC curves were performed and the AUCs of the 0.5-, 1.0-, and 1.5-year survival for the constructed nomogram in the training cohort were 0.737, 0.673, and 0.694, respectively, in the validation cohort (Figure 6B). The nomogram shared a C-index of more than 0.6 at different times (Figure 6C) and a relatively low prediction error (Figure 6D). Finally, the calibration curves for this nomogram in the validation cohort at 0.5 year was poor, while those at 1 year and 1.5 years were good (Figures 6E–G).

### Differential Abundances of Infiltrative Immune Cells

**FIGURE 7:**
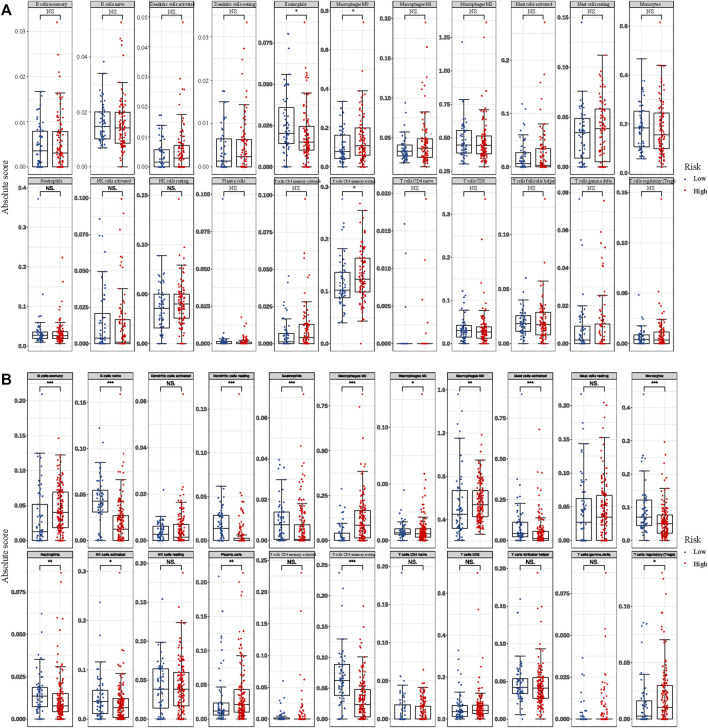
Differential abundances of infiltrative immune cells between high- and low-RCN1 groups. **(A)** TCGA. **(B)** CGGA.

By using the CIBERSORTx algorithm, the relative proportions of 22 immune cells between two groups in GBM were obtained. In the TCGA dataset, there were three types of infiltrative immune cells with significant difference at different groups, whereas in CGGA there was fourteen, in which T cells CD4 memory resting, eosinophils, and macrophages M0 were differentially expressed in two data at the same time (Figure 7). With more detail as shown by bar plots in Figure 7A, in the training cohort, the infiltration level of eosinophils was significantly higher in the low-risk group, whereas the infiltration level of macrophages M0 and T cells CD4 memory resting was significantly higher in the high-risk group. In the validation cohort, the infiltration levels of B cells naive, dendritic cells resting, mast cells activated, macrophages M1, eosinophils, T cells CD4 memory resting, neutrophils, NK cells activated, and monocytes were significantly higher in the low-risk group, whereas the B cells memory, macrophages (M0, M2), T cells regulatory (Tregs), and plasma cells were significantly higher in the high-risk group (Figure 7B).

### Pathway Enrichment Analysis

**FIGURE 8:**
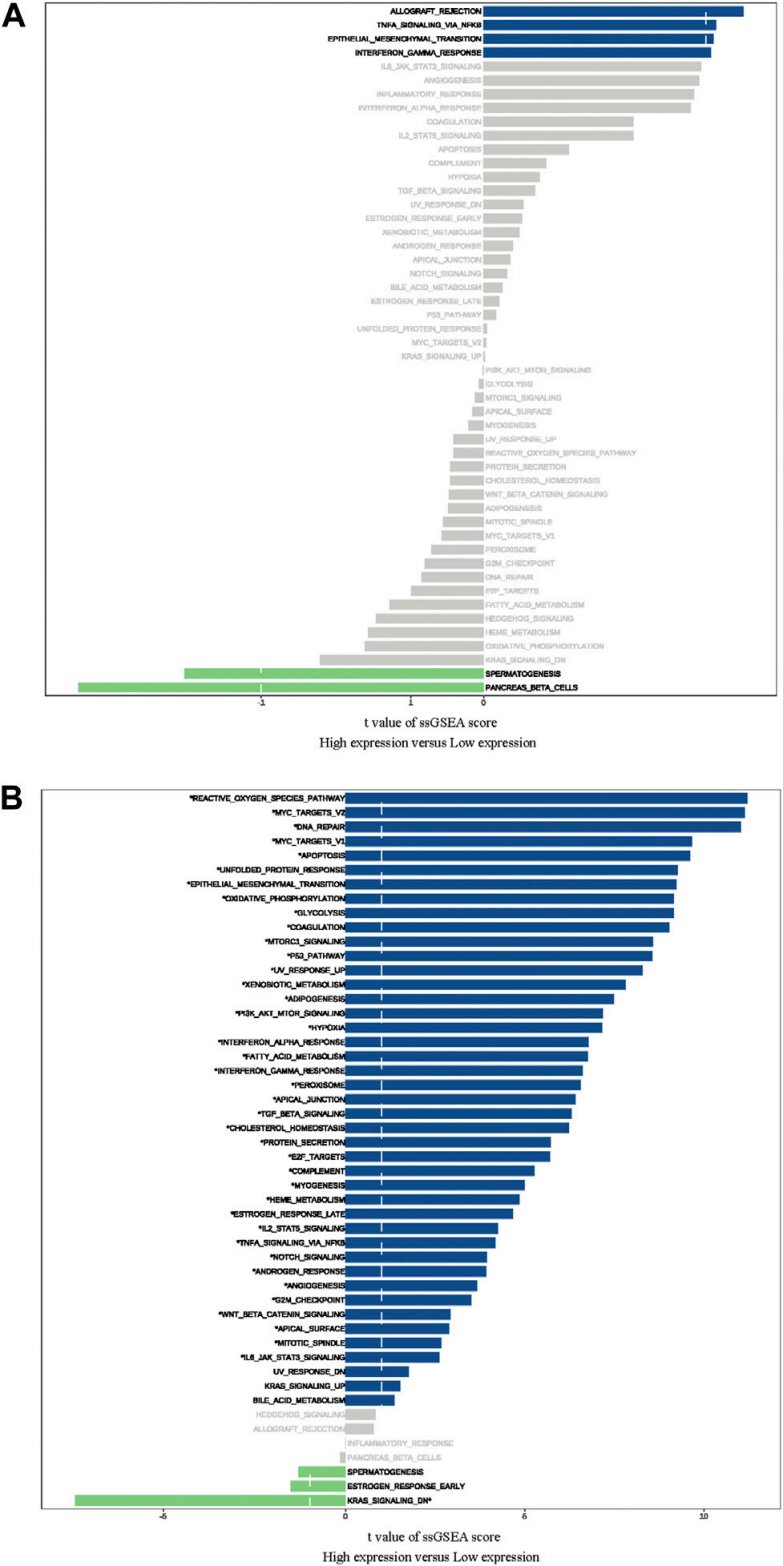
Difference of Hallmark gene set in high- and low-RCN1 groups. **(A)** TCGA. **(B)** CGGA.

The ssGSEA was used to identify signaling pathways RCN1-involved in GBM, and then demonstrate significant differences in the enrichment of MSigDB hallmark gene set in the TCGA and CGGA databases, respectively. The results indicated that there was no significant pathway screened in the TCGA database (Figure 8A), whereas the KRAS-signaling-DN pathway was significantly involved in the low-RCN1 group and some pathways, including reactive oxygen species, MYC targets V2 and V1, apoptosis, and DNA repair pathways, in the high-RCN1 group in the CGGA database (Figure 8B).

### Knockdown of Reticulocalbin-1Inhibited Cell Invasion in Glioblastoma Multiforme

**FIGURE 9:**
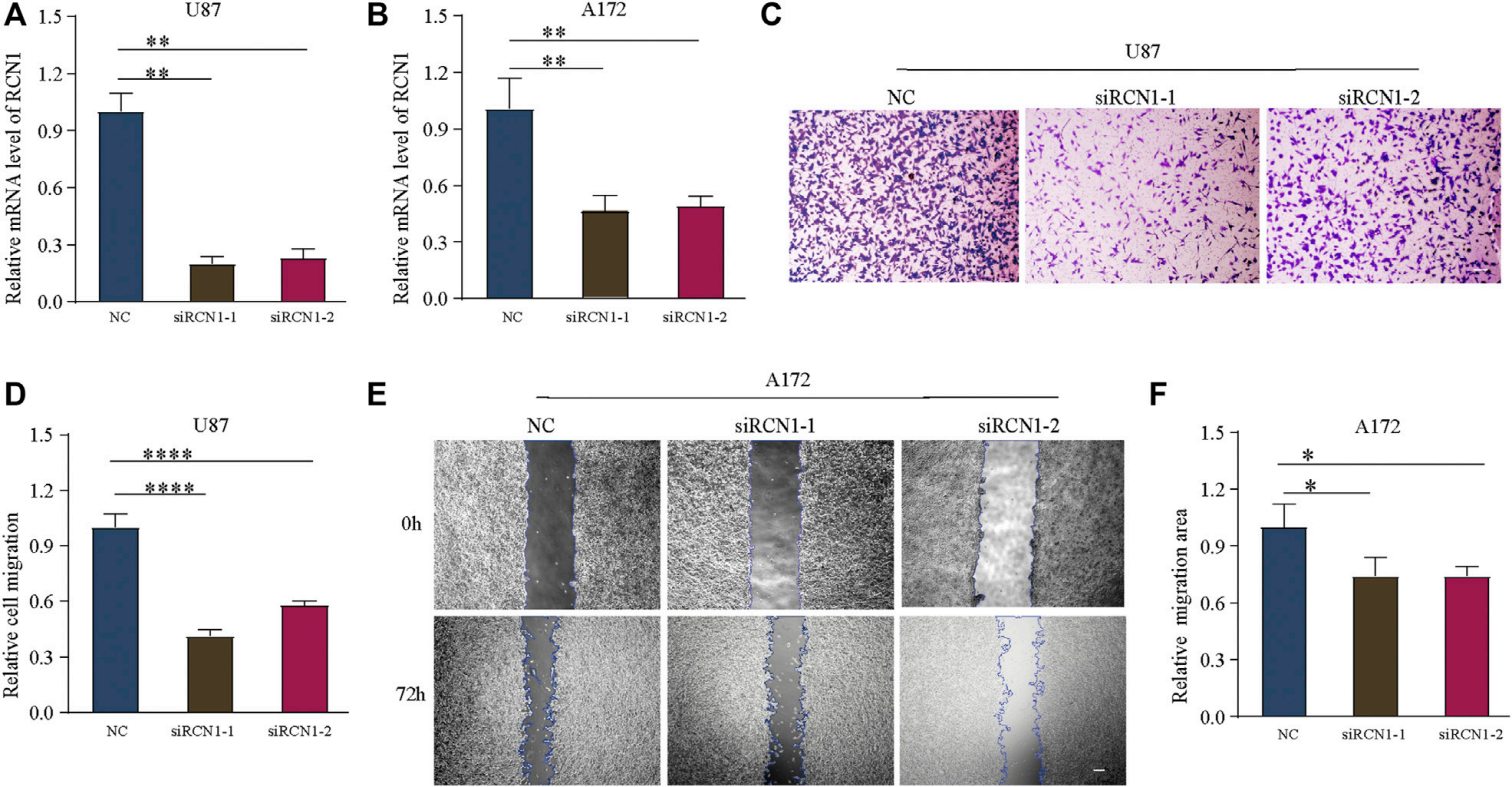
Down-expression of RCN1 inhibits cell migration. **(A,B)** The mRNA of RCN1 knockdown on U87 and A172 cells after transfection. **(C,D)** Transwell assay. **(E,F)** Would healing assay.

Finally, in order to elucidate the effect of RCN1on GBM cell invasion, we conducted a series of morphological and molecular biological experiments. The si-RCN1-1 and si-RCN1-2 could effectively reduce endogenous RCN1 mRNA expression by mRNA levels in both U87 and A172 cells (Figures 9A,B). Later, we confirmed that si-RCN1 could decrease cell invasion (Figures 9C–F).

## Discussion

GBM is the most aggressive brain tumor. The prognosis of patients with GBM remains poor, although treatment strategies, including maximum surgical resection, radiation, and chemotherapy, have been conducted. The role and mechanism of biomarkers in GBM tumorigenesis are very important for the development of treatment of patients with GBM (Sasmita et al., 2018). Recently, ample molecular markers have been identified, which could provide new insight regarding GBM formation and progression. The discoveries and roles of molecular biomarkers, GBM-specific microRNAs, and GBM stem cells were delineated (Sasmita et al., 2018; Hassn Mesrati et al., 2020). There are some genetic mutation features, epigenetic modification, and some molecular alterations *in situ*. As highlighted by Alireza Mansouri and his colleagues, the methylation of *MGMT* promoter has been identified to provide better outcome prediction when GBM patients receive temozolomide chemotherapy (Mansouri et al., 2019). Meanwhile, the amplification of *CCND2* suggests better clinical outcomes in *IDH*-mutant patients while the elevated total copy number variation, co-amplification of *CDK4/MDM2*, amplification of *PDGFRA*, and *CDKN2A* show worse association (Zheng et al., 2013; Sasmita et al., 2018; Mirchia et al., 2019; Louis et al., 2021). Large-scale studies further demonstrated that *IDH*-mutant GBMs tend to have a higher concentration of 2-hydroxyglutarate (an oncometabolite produced as a result of the *IDH1*/*2* mutation), which may promote infiltration via upregulation of HIF-1α and VEGF(K. Mirchia and Richardson, 2020; Sasmita et al., 2018). For IDH-wide type GBM patients, the mutation of *BRAF* may benefit them while the co-alteration of *EGFR/PTEN/CDKN2A* and mutation of *PIK3CA* and *H3K27M* correlates with worse clinical outcomes (Mirchia and Richardson, 2020; Umehara et al., 2019). Circulating biomarkers have also been developed in GBM because of their non-invasive potential (Jelski and Mroczko, 2021; Kefayat et al., 2021), but the sensitivity and specificity are still problems to be solved (Müller Bark et al., 2020; Raza et al., 2020; Jones et al., 2021). Additionally, there are still no clinically validated circulating biomarkers for GBM patients because of the limitation in blood-brain-barrier, low concentration, and their short half-life (Müller Bark et al., 2020). It could not even be proven the release of potential biomarkers were exclusive from tumor cells or not. According to a systematic review, about 133 distinct biomarkers were identified from 1853 patients and evaluated based on level of evidence (IA∼IVD) using an adapted framework from the National Comprehensive Cancer Network guideline (Raza et al., 2020). Nevertheless, few can reach level IA and further refinements are needed. Therefore, it is urgent to identify new biomarkers that can illustrate the in-depth molecular mechanism, enhance the diagnosis, respond to treatment, and provide prognostic prediction of GBM. Here, we identified the potential of RCN in GBM. RCN1, as a calcium-binding protein located in the lumen of the endoplasmic reticulum, containing six conserved regions with similarity to a high-affinity calcium-binding motif, the EF-hand. Recently, several studies have revealed that RCN1 acts as an oncogene involved in tumor progression. In our study, we found that the expression of RCN1 is higher in GBM samples than normal brain tissues by utilizing the GEPIA2 online tool, which is consistent with previous findings that high RCN1 expression is present in various malignancies (Amatschek et al., 2004). Recently, RCN1 has been demonstrated to be a prognostic marker in various cancers, such as non-small cell lung cancer (Chen et al., 2019) and renal cell carcinoma (Giribaldi et al., 2013). However, the function and the underlying mechanism of RCN1 involved in GBM remains a vague notion. Based on the observation in previous studies (Chen et al., 2019; Giribaldi et al., 2013; Liu et al., 2018), we hypothesized that RCN1 could be a promising prognostic marker or as a response predictor for targeted therapy in GBM. Interestingly, we confirmed our hypothesis through TCGA and validated them in CGGA and experiments. At the same time, the mechanisms behind RCN1 were also explored and the identified pathways were consistent with previous studies (Fukasawa et al., 2021; Pucci et al., 2021; Sighel et al., 2021; Xue et al., 2021).

The convenient access to the public database allows us for the application of large-scale gene expression profiling and database mining for potential correlation between genes and overall survival of a variety of malignancies including GBM (Aldape et al., 2015; Zhou et al., 2021). In the present study, RCN1 was identified as an independent factor for the prognosis of GBM patients in TCGA, according to the results of univariate and multivariate analysis, with or without adjustment for confounders on the survival difference as described in a previous study (Erturk and Tas, 2017). By identifying the confounding factors, we further revealed the independent prognostic role of RCN1 in GBM patients. Since several clinical characteristics have been identified to influence the outcomes of GBM patients, interaction may exist (Brankovic et al., 2019) and it is plausible to perform subgroup analysis to estimate the interaction between RCN1 and selective clinical characteristics (Gönen, 2003). Here we innovatively identified confounding factors to increase the reliability of the results. Discrimination and calibration are the most commonly used indicators in evaluating prediction models. However, a systematic review found that 63% of the studies reported the discrimination information of prediction models, but only 36% of the studies reported the calibration information (Wessler et al., 2015); we reported both discrimination and calibration.

Recently, endoplasmic reticulum (ER) stress has been reported to have a considerable impact on cell growth, proliferation, metastasis, invasion, angiogenesis, and chemoradiotherapy resistance in various cancers. Accumulated studies have demonstrated that ER stress governs multiple pro-tumoural attributes in the cancer cell mainly via reprogramming the function of immune cells. The tumor microenvironment can be shaped because of the functional impact of ER stress responses in endothelial cells, cancer-associated fibroblasts, and other stromal cells during cancer progression (Chen and Cubillos-Ruiz, 2021). ER stress and downstream autophagy in the regulation of cell fate may function in temozolomide treatment and they have the potential to be therapeutic targets in GBM (He et al., 2019). Besides, it was revealed that the ER stress-related genes-based risk model can serve as a prognostic factor to predict the outcome for patients and be correlated with immune and inflammation responses in glioma (Zhang et al., 2021). Among these genes, RCN1, as an ER-resident calcium-binding protein, is verified as one of ER stress-related genes, of which the depletion causes the ER stress-induced cells’ apoptosis in various cancers (Huang et al., 2020; Liu et al., 2018; Xu et al., 2017). In the early study, it was demonstrated that RCN1 was identified as a genuine phagocytosis ligand to stimulate microglial phagocytosis of apoptotic neurons that were subsequently targeted by phagosomes (Ding et al., 2015). It was also observed that RCN1 interacts with SEC63p to activate protein translocation and quality control pathways in the ER (Honoré, 2009). As mentioned above, RCN1 proteins can not only have intrinsic roles in tumor development but also serve as a regulator involved in immune-related activities. Considering the powerful role of tumor microenvironment cells contributing significantly to prognosis, we further investigated the immune cell infiltration between the high- and low-risk groups in the TCGA and CGGA datasets, respectively. It was revealed that the infiltration levels of T cells CD4 memory resting, eosinophils, and macrophages M0 were differentially changed in both TCGA and CGGA cohorts. Furthermore, we found that macrophages M0 was significantly higher in the high-risk group both in the TCGA and CGGA datasets, representing a certain relationship between them.

Furthermore, our results also suggested that high RCN1 may have a certain biological function in GBM. Therefore, to further investigate the role RCN1 played in GBM, we performed *in vitro* experiments and verified that the downregulation of RCN1 inhibited the cell invasion in GBM cell lines. The results of functional experiments are consistent with the above mentioned in our study, and further confirmed the critical role of RCN1 in GBM.

Several limitations should be noted in our study. Firstly, although we selected clinical samples from two different databases for mutual validation, verification in a larger sample is needed in the future. In addition, the variables involved in our prognostic model are easy to obtain, which is undoubtedly very convenient for clinical application, but it also needs to be further confirmed in clinical practice. Thirdly, although we verified the results of rigorous data mining, more animal or clinical exploration is still urgently needed. In addition, as for the absence of 1p19q characterization in TCGA, the status of 1p19q codeletion was not included for analyses. Finally, considering a lack of verification from clinical samples, the effectiveness of the RCN1-signature in GBM patients needs to be further verified by well-designed investigations in clinic. And how RCN1 regulates ER stress biology still needs to be further illustrated by experiments.

Overall, we found that high RCN1 has a poor OS for GBM patients, and confirmed RCN1 as an independent prognostic factor and developed a prognostic predictive model based on RCN1, which performed well in the prediction of OS for GBM patients.

## Data Availability

The original contributions presented in the study are included in the article/Supplementary Material, further inquiries can be directed to the corresponding authors.

## Supplementary Material

Supplementary Figure S1 RCN1 is an independent prognostic signature compared with others. Different potential prognostic signatures were evaluated based on the data from TCGA and CCGA using the ROC curve.

## References

[B1] Aldape K. Zadeh G. Mansouri S. Reifenberger G. von Deimling A. (2015). Glioblastoma: Pathology, Molecular Mechanisms and Markers. Acta Neuropathol. 129 (6), 829–848. 10.1007/s00401-015-1432-1 25943888

[B2] Ali H. R. Chlon L. Pharoah P. D. P. Markowetz F. Caldas C. (2016). Patterns of Immune Infiltration in Breast Cancer and Their Clinical Implications: A Gene-Expression-Based Retrospective Study. Plos Med. 13 (12), e1002194. 10.1371/journal.pmed.1002194 27959923 PMC5154505

[B3] Amatschek S. Koenig U. Auer H. Steinlein P. Pacher M. Gruenfelder A. (2004). Tissue-Wide Expression Profiling Using cDNA Subtraction and Microarrays to Identify Tumor-Specific Genes. Cancer Res. 64 (3), 844–856. 10.1158/0008-5472.can-03-2361 14871811

[B4] Barbie D. A. Tamayo P. Boehm J. S. Kim S. Y. Moody S. E. Dunn I. F. (2009). Systematic RNA Interference Reveals that Oncogenic KRAS-Driven Cancers Require TBK1. Nature 462 (7269), 108–112. 10.1038/nature08460 19847166 PMC2783335

[B5] Blanche P. Dartigues J.-F. Jacqmin-Gadda H. (2013). Estimating and Comparing Time-Dependent Areas under Receiver Operating Characteristic Curves for Censored Event Times with Competing Risks. Statist. Med. 32 (30), 5381–5397. 10.1002/sim.5958 24027076

[B6] Brankovic M. Kardys I. Steyerberg E. W. Lemeshow S. Markovic M. Rizopoulos D. (2019). Understanding of Interaction (Subgroup) Analysis in Clinical Trials. Eur. J. Clin. Invest. 49 (8), e13145. 10.1111/eci.13145 31135965

[B7] Chen X. Cubillos-Ruiz J. R. (2021). Endoplasmic Reticulum Stress Signals in the Tumour and its Microenvironment. Nat. Rev. Cancer 21 (2), 71–88. 10.1038/s41568-020-00312-2 33214692 PMC7927882

[B8] Chen X. Shao W. Huang H. Feng X. Yao S. Ke H. (2019). Overexpression of RCN1 Correlates with Poor Prognosis and Progression in Non-Small Cell Lung Cancer. Hum. Pathol. 83, 140–148. 10.1016/j.humpath.2018.08.014 30172915

[B9] Ding Y. Caberoy N. B. Guo F. LeBlanc M. E. Zhang C. Wang W. (2015). Reticulocalbin-1 Facilitates Microglial Phagocytosis. PLoS One 10 (5), e0126993. 10.1371/journal.pone.0126993 25992960 PMC4436338

[B10] Erturk K. Tas F. (2017). Effect of Biology on the Outcome of Female Melanoma Patients. Mol. Clin. Onc 7 (6), 1093–1100. 10.3892/mco.2017.1446 PMC5740849 29285381

[B11] Fukasawa K. Kadota T. Horie T. Tokumura K. Terada R. Kitaguchi Y. (2021). CDK8 Maintains Stemness and Tumorigenicity of Glioma Stem Cells by Regulating the C-MYC Pathway. Oncogene 40 (15), 2803–2815. 10.1038/s41388-021-01745-1 33727660

[B12] Gilbert M. R. Wang M. Aldape K. D. Stupp R. Hegi M. E. Jaeckle K. A. (2013). Dose-Dense Temozolomide for Newly Diagnosed Glioblastoma: A Randomized Phase III Clinical Trial. J. Clin. Oncol. 31 (32), 4085–4091. 10.1200/jco.2013.49.6968 24101040 PMC3816958

[B13] Giribaldi G. Barbero G. Mandili G. Daniele L. Khadjavi A. Notarpietro A. (2013). Proteomic Identification of Reticulocalbin 1 as Potential Tumor Marker in Renal Cell Carcinoma. J. Proteomics 91, 385–392. 10.1016/j.jprot.2013.07.018 23916412

[B14] Gönen M. (2003). Planning for Subgroup Analysis: A Case Study of Treatment-Marker Interaction in Metastatic Colorectal Cancer. Controlled Clin. Trials 24 (4), 355–363. 10.1016/s0197-2456(03)00006-0 12865031

[B15] Grzeskowiak R. Witt H. Drungowski M. Thermann R. Hennig S. Perrot A. (2003). Expression Profiling of Human Idiopathic Dilated Cardiomyopathy. Cardiovasc. Res. 59 (2), 400–411. 10.1016/s0008-6363(03)00426-7 12909323

[B16] GTEx Consortium (2015). Human Genomics. The Genotype-Tissue Expression (GTEx) Pilot Analysis: Multitissue Gene Regulation in Humans. Science 348 (6235), 648–660. 10.1126/science.1262110 25954001 PMC4547484

[B17] Hainfellner J. Louis D. N. Perry A. Wesseling P. (2014). Letter in Response to David N. Louiset Al, International Society of Neuropathology-Haarlem Consensus Guidelines for Nervous System Tumor Classification and Grading, Brain Pathology, Doi: 10.1111/bpa.12171. Brain Pathol. 24 (6), 671–672. 10.1111/bpa.12187 25345897 PMC9426753

[B18] Harrell F. E. Jr. Lee K. L. Mark D. B. (1996). Multivariable Prognostic Models: Issues in Developing Models, Evaluating Assumptions and Adequacy, and Measuring and Reducing Errors. Stat. Med. 15 (4), 361–387. 10.1002/(SICI)1097-0258(19960229)15:4<361::AID-SIM168>3.0.CO;2-4 8668867

[B19] Hartmann C. Hentschel B. Wick W. Capper D. Felsberg J. Simon M. (2010). Patients with IDH1 Wild Type Anaplastic Astrocytomas Exhibit Worse Prognosis Than IDH1-Mutated Glioblastomas, and IDH1 Mutation Status Accounts for the Unfavorable Prognostic Effect of Higher Age: Implications for Classification of Gliomas. Acta Neuropathol. 120 (6), 707–718. 10.1007/s00401-010-0781-z 21088844

[B20] Hassn Mesrati M. Behrooz A. B. Y. Abuhamad A. Syahir A. (2020). Understanding Glioblastoma Biomarkers: Knocking a Mountain with a Hammer. Cells 9 (5), 1236. 10.3390/cells9051236 PMC7291262 32429463

[B21] He Y. Su J. Lan B. Gao Y. Zhao J. (2019). Targeting Off-Target Effects: Endoplasmic Reticulum Stress and Autophagy as Effective Strategies to Enhance Temozolomide Treatment. OncoTargets Ther. 12, 1857–1865. 10.2147/OTT.S194770 PMC6413742 30881038

[B22] Heagerty P. J. Lumley T. Pepe M. S. (2000). Time-Dependent ROC Curves for Censored Survival Data and a Diagnostic Marker. Biometrics 56 (2), 337–344. 10.1111/j.0006-341x.2000.00337.x 10877287

[B23] Honoré B. (2009). The Rapidly Expanding CREC Protein Family: Members, Localization, Function, and Role in Disease. BioEssays 31 (3), 262–277. 10.1002/bies.200800186 19260022

[B24] Huang Z.-H. Qiao J. Feng Y.-Y. Qiu M.-T. Cheng T. Wang J. (2020). Reticulocalbin-1 Knockdown Increases the Sensitivity of Cells to Adriamycin in Nasopharyngeal Carcinoma and Promotes Endoplasmic Reticulum Stress-Induced Cell Apoptosis. Cell Cycle 19 (13), 1576–1589. 10.1080/15384101.2020.1733750 32436770 PMC7469451

[B25] Jayamanne D. Wheeler H. Cook R. Teo C. Brazier D. Schembri G. (2018). Survival Improvements with Adjuvant Therapy in Patients with Glioblastoma. ANZ J. Surg. 88 (3), 196–201. 10.1111/ans.14153 28922698

[B26] Jelski W. Mroczko B. (2021). Molecular and Circulating Biomarkers of Brain Tumors. Int. J. Mol. Sci. 22 (13), 7039. 10.3390/ijms22137039 34210107 PMC8268709

[B27] Jones J. Nguyen H. Drummond K. Morokoff A. (2021). Circulating Biomarkers for Glioma: A Review. Neurosurgery 88 (3), E221–E230. 10.1093/neuros/nyaa540 33442748

[B28] Kefayat A. Amouheidari A. Ghahremani F. Alirezaei Z. (2021). Diagnostic and Prognostic Value of Stem Cell Factor Plasma Level in Glioblastoma Multiforme Patients. Cancer Med. 10 (15), 5154–5162. 10.1002/cam4.4073 34250760 PMC8335833

[B29] Kernan W. N. Viscoli C. M. Brass L. M. Broderick J. P. Brott T. Feldmann E. (2000). Phenylpropanolamine and the Risk of Hemorrhagic Stroke. N. Engl. J. Med. 343 (25), 1826–1832. 10.1056/nejm200012213432501 11117973

[B30] Kerr K. F. Brown M. D. Zhu K. Janes H. (2016). Assessing the Clinical Impact of Risk Prediction Models with Decision Curves: Guidance for Correct Interpretation and Appropriate Use. J. Clin. Oncol. 34 (21), 2534–2540. 10.1200/JCO.2015.65.5654 27247223 PMC4962736

[B31] Liang B. Liang Y. Li R. Zhang H. Gu N. (2021). Integrating Systematic Pharmacology-Based Strategy and Experimental Validation to Explore the Synergistic Pharmacological Mechanisms of Guanxin V in Treating Ventricular Remodeling. Bioorg. Chem. 115, 105187. 10.1016/j.bioorg.2021.105187 34303037

[B32] Liberzon A. Birger C. Thorvaldsdóttir H. Ghandi M. Mesirov J. P. Tamayo P. (2015). The Molecular Signatures Database Hallmark Gene Set Collection. Cel Syst. 1 (6), 417–425. 10.1016/j.cels.2015.12.004 PMC4707969 26771021

[B33] Liu X. Zhang N. Wang D. Zhu D. Yuan Q. Zhang X. (2018). Downregulation of Reticulocalbin‐1 Differentially Facilitates Apoptosis and Necroptosis in Human Prostate Cancer Cells. Cancer Sci. 109 (4), 1147–1157. 10.1111/cas.13541 29453900 PMC5891187

[B34] Liu Z. Brattain M. G. Appert H. (1997). Differential Display of Reticulocalbin in the Highly Invasive Cell Line, MDA-MB-435, Versus the Poorly Invasive Cell Line, MCF-7. Biochem. Biophysical Res. Commun. 231 (2), 283–289. 10.1006/bbrc.1997.6083 9070264

[B35] Louis D. N. Perry A. Reifenberger G. von Deimling A. Figarella-Branger D. Cavenee W. K. (2016). The 2016 World Health Organization Classification of Tumors of the Central Nervous System: a Summary. Acta Neuropathol. 131 (6), 803–820. 10.1007/s00401-016-1545-1 27157931

[B36] Louis D. N. Perry A. Wesseling P. Brat D. J. Cree I. A. Figarella-Branger D. (2021). The 2021 WHO Classification of Tumors of the Central Nervous System: A Summary. Neuro Oncol. 23 (8), 1231–1251. 10.1093/neuonc/noab106 34185076 PMC8328013

[B37] Lu J.-J. Lu D.-Z. Chen Y.-F. Dong Y.-T. Zhang J.-R. Li T. (2015). Proteomic Analysis of Hepatocellular Carcinoma HepG2 Cells Treated with Platycodin D. Chin. J. Nat. Medicines 13 (9), 673–679. 10.1016/s1875-5364(15)30065-0 26412427

[B38] Mansouri A. Hachem L. D. Mansouri S. Nassiri F. Laperriere N. J. Xia D. (2019). MGMT Promoter Methylation Status Testing to Guide Therapy for Glioblastoma: Refining the Approach Based on Emerging Evidence and Current Challenges. Neuro Oncol. 21 (2), 167–178. 10.1093/neuonc/noy132 30189035 PMC6374759

[B39] Mirchia K. Richardson T. E. (2020). Beyond IDH-Mutation: Emerging Molecular Diagnostic and Prognostic Features in Adult Diffuse Gliomas. Cancers 12 (7), 1817. 10.3390/cancers12071817 PMC7408495 32640746

[B40] Mirchia K. Sathe A. A. Walker J. M. Fudym Y. Galbraith K. Viapiano M. S. (2019). Total Copy Number Variation as a Prognostic Factor in Adult Astrocytoma Subtypes. Acta Neuropathol. Commun. 7 (1), 92. 10.1186/s40478-019-0746-y 31177992 PMC6556960

[B41] Müller Bark J. Kulasinghe A. Chua B. Day B. W. Punyadeera C. (2020). Circulating Biomarkers in Patients with Glioblastoma. Br. J. Cancer 122 (3), 295–305. 10.1038/s41416-019-0603-6 31666668 PMC7000822

[B42] Nakakido M. Tamura K. Chung S. Ueda K. Fujii R. Kiyotani K. (2016). Phosphatidylinositol Glycan Anchor Biosynthesis, Class X Containing Complex Promotes Cancer Cell Proliferation through Suppression of EHD2 and ZIC1, Putative Tumor Suppressors. Int. J. Oncol. 49 (3), 868–876. 10.3892/ijo.2016.3607 27572108 PMC4948962

[B43] Newman A. M. Steen C. B. Liu C. L. Gentles A. J. Chaudhuri A. A. Scherer F. (2019). Determining Cell Type Abundance and Expression from Bulk Tissues with Digital Cytometry. Nat. Biotechnol. 37 (7), 773–782. 10.1038/s41587-019-0114-2 31061481 PMC6610714

[B44] Nimmrich I. Erdmann S. Melchers U. Finke U. Hentsch S. Moyer M. P. (2000). Seven Genes that Are Differentially Transcribed in Colorectal Tumor Cell Lines. Cancer Lett. 160 (1), 37–43. 10.1016/s0304-3835(00)00553-x 11098082

[B45] Ogłuszka M. Orzechowska M. Jędroszka D. Witas P. Bednarek A. K. (2019). Evaluate Cutpoints: Adaptable Continuous Data Distribution System for Determining Survival in Kaplan-Meier Estimator. Comput. Methods Programs Biomed. 177, 133–139. 10.1016/j.cmpb.2019.05.023 31319941

[B46] Omuro A. DeAngelis L. M. (2013). Glioblastoma and Other Malignant Gliomas. Jama 310 (17), 1842–1850. 10.1001/jama.2013.280319 24193082

[B47] Ostrom Q. T. Gittleman H. Stetson L. Virk S. M. Barnholtz-Sloan J. S. (2015). Epidemiology of Gliomas. Cancer Treat. Res. 163, 1–14. 10.1007/978-3-319-12048-5_1 25468222

[B48] Ozawa M. Muramatsu T. (1993). Reticulocalbin, a Novel Endoplasmic Reticulum Resident Ca(2+)-Binding Protein with Multiple EF-Hand Motifs and a Carboxyl-Terminal HDEL Sequence. J. Biol. Chem. 268 (1), 699–705. Retrieved from: http://www.ncbi.nlm.nih.gov/pubmed/8416973 . 10.1016/s0021-9258(18)54208-3 8416973

[B49] Pencina M. J. D' Agostino R. B. D' Agostino. Vasan R. S. Jr. Vasan R. S. (2008). Evaluating the Added Predictive Ability of a New Marker: From Area under the ROC Curve to Reclassification and beyond. Statist. Med. 27 (2), 157–172. discussion 207-112. 10.1002/sim.2929 17569110

[B50] Pucci C. Marino A. Şen Ö. De Pasquale D. Bartolucci M. Iturrioz-Rodríguez N. (2021). Ultrasound-Responsive Nutlin-Loaded Nanoparticles for Combined Chemotherapy and Piezoelectric Treatment of Glioblastoma Cells. Acta Biomater. S1742-7061 (21), 00242–00247. 10.1016/j.actbio.2021.04.005 PMC7612320 33894347

[B51] Raza I. J. Tingate C. A. Gkolia P. Romero L. Tee J. W. Hunn M. K. (2020). Blood Biomarkers of Glioma in Response Assessment Including Pseudoprogression and Other Treatment Effects: A Systematic Review. Front. Oncol. 10, 1191. 10.3389/fonc.2020.01191 32923382 PMC7456864

[B52] Rousson V. Zumbrunn T. (2011). Decision Curve Analysis Revisited: Overall Net Benefit, Relationships to ROC Curve Analysis, and Application to Case-Control Studies. BMC Med. Inform. Decis. Mak 11, 45. 10.1186/1472-6947-11-45 21696604 PMC3148204

[B53] Sasmita A. O. Wong Y. P. Ling A. P. K. (2018). Biomarkers and Therapeutic Advances in Glioblastoma Multiforme. Asia-pac J. Clin. Oncol. 14 (1), 40–51. 10.1111/ajco.12756 28840962

[B54] Sighel D. Notarangelo M. Aibara S. Re A. Ricci G. Guida M. (2021). Inhibition of Mitochondrial Translation Suppresses Glioblastoma Stem Cell Growth. Cel Rep. 35 (4), 109024. 10.1016/j.celrep.2021.109024 PMC8097689 33910005

[B55] Soffietti R. Bertero L. Pinessi L. Rudà R. (2014). Pharmacologic Therapies for Malignant Glioma: A Guide for Clinicians. CNS Drugs 28 (12), 1127–1137. 10.1007/s40263-014-0215-x 25403944

[B56] Soria J.-C. Felip E. Cobo M. Lu S. Syrigos K. Lee K. H. (2015). Afatinib versus Erlotinib as Second-Line Treatment of Patients with Advanced Squamous Cell Carcinoma of the Lung (LUX-Lung 8): An Open-Label Randomised Controlled Phase 3 Trial. Lancet Oncol. 16 (8), 897–907. 10.1016/s1470-2045(15)00006-6 26156651

[B57] Subramanian A. Tamayo P. Mootha V. K. Mukherjee S. Ebert B. L. Gillette M. A. (2005). Gene Set Enrichment Analysis: A Knowledge-Based Approach for Interpreting Genome-Wide Expression Profiles. Proc. Natl. Acad. Sci. 102 (43), 15545–15550. 10.1073/pnas.0506580102 16199517 PMC1239896

[B58] Tang Z. Kang B. Li C. Chen T. Zhang Z. (2019). GEPIA2: An Enhanced Web Server for Large-Scale Expression Profiling and Interactive Analysis. Nucleic Acids Res. 47 (W1), W556–w560. 10.1093/nar/gkz430 31114875 PMC6602440

[B59] Tang Z. Li C. Kang B. Gao G. Li C. Zhang Z. (2017). GEPIA: A Web Server for Cancer and Normal Gene Expression Profiling and Interactive Analyses. Nucleic Acids Res. 45 (W1), W98–w102. 10.1093/nar/gkx247 28407145 PMC5570223

[B60] Umehara T. Arita H. Yoshioka E. Shofuda T. Kanematsu D. Kinoshita M. (2019). Distribution Differences in Prognostic Copy Number Alteration Profiles in IDH-Wild-Type Glioblastoma Cause Survival Discrepancies across Cohorts. Acta Neuropathol. Commun. 7 (1), 99. 10.1186/s40478-019-0749-8 31215469 PMC6580599

[B61] Vickers A. J. Cronin A. M. Elkin E. B. Gonen M. (2008). Extensions to Decision Curve Analysis, a Novel Method for Evaluating Diagnostic Tests, Prediction Models and Molecular Markers. BMC Med. Inform. Decis. Mak 8, 53. 10.1186/1472-6947-8-53 19036144 PMC2611975

[B62] Vickers A. J. Elkin E. B. (2006). Decision Curve Analysis: A Novel Method for Evaluating Prediction Models. Med. Decis. Making 26 (6), 565–574. 10.1177/0272989x06295361 17099194 PMC2577036

[B63] Wessler B. S. Lai Yh L. Kramer W. Cangelosi M. Raman G. Lutz J. S. (2015). Clinical Prediction Models for Cardiovascular Disease. Circ. Cardiovasc. Qual. Outcomes 8 (4), 368–375. 10.1161/circoutcomes.115.001693 26152680 PMC4512876

[B64] Wick W. Meisner C. Hentschel B. Platten M. Schilling A. Wiestler B. (2013). Prognostic or Predictive Value of MGMT Promoter Methylation in Gliomas Depends on IDH1 Mutation. Neurology 81 (17), 1515–1522. 10.1212/WNL.0b013e3182a95680 24068788

[B65] Xu S. Xu Y. Chen L. Fang Q. Song S. Chen J. (2017). RCN1 Suppresses ER Stress-Induced Apoptosis via Calcium Homeostasis and PERK-CHOP Signaling. Oncogenesis 6 (3), e304. 10.1038/oncsis.2017.6 28319095 PMC5533947

[B66] Xue Y. Fu Y. Zhao F. Gui G. Li Y. Rivero-Hinojosa S. (2021). Frondoside A Inhibits an MYC-Driven Medulloblastoma Model Derived from Human-Induced Pluripotent Stem Cells. Mol. Cancer Ther. 20, 1199–1209. 10.1158/1535-7163.MCT-20-0603 33722850 PMC8172454

[B67] Zhang Q. Guan G. Cheng P. Cheng W. Yang L. Wu A. (2021). Characterization of an Endoplasmic Reticulum Stress‐related Signature to Evaluate Immune Features and Predict Prognosis in Glioma. J. Cel Mol Med 25, 3870–3884. 10.1111/jcmm.16321 PMC8051731 33611848

[B68] Zhang Y. Ye J. Chen D. Zhao X. Xiao X. Tai S. (2006). Differential Expression Profiling between the Relative normal and Dystrophic Muscle Tissues from the Same LGMD Patient. J. Transl Med. 4, 53. 10.1186/1479-5876-4-53 17176482 PMC1769400

[B69] Zheng S. Fan J. Yu F. Feng B. Lou B. Zou Q. (2020). Viral Load Dynamics and Disease Severity in Patients Infected with SARS-CoV-2 in Zhejiang Province, China, January-March 2020: Retrospective Cohort Study. BMJ 369, m1443. 10.1136/bmj.m1443 32317267 PMC7190077

[B70] Zheng S. Fu J. Vegesna R. Mao Y. Heathcock L. E. Torres-Garcia W. (2013). A Survey of Intragenic Breakpoints in Glioblastoma Identifies a Distinct Subset Associated with Poor Survival. Genes Develop. 27 (13), 1462–1472. 10.1101/gad.213686.113 23796897 PMC3713427

[B71] Zhou J.-G. Liang B. Jin S.-H. Liao H.-L. Du G.-B. Cheng L. (2019). Development and Validation of an RNA-Seq-Based Prognostic Signature in Neuroblastoma. Front. Oncol. 9, 1361. 10.3389/fonc.2019.01361 31867276 PMC6904333

[B72] Zhou J.-G. Liang B. Liu J.-G. Jin S.-H. He S.-S. Frey B. (2021). Identification of 15 lncRNAs Signature for Predicting Survival Benefit of Advanced Melanoma Patients Treated with Anti-PD-1 Monotherapy. Cells 10 (5), 977. 10.3390/cells10050977 33922038 PMC8143567

