# Supplementary figures and images for: Integrative Analyses and Verification of the Expression and Prognostic Significance for RCN1 in Glioblastoma Multiforme
Source: Front Mol Biosci. 2021 Oct 13;8:736947. doi: 10.3389/fmolb.2021.736947 (PMC8548715; doi:10.3389/fmolb.2021.736947)

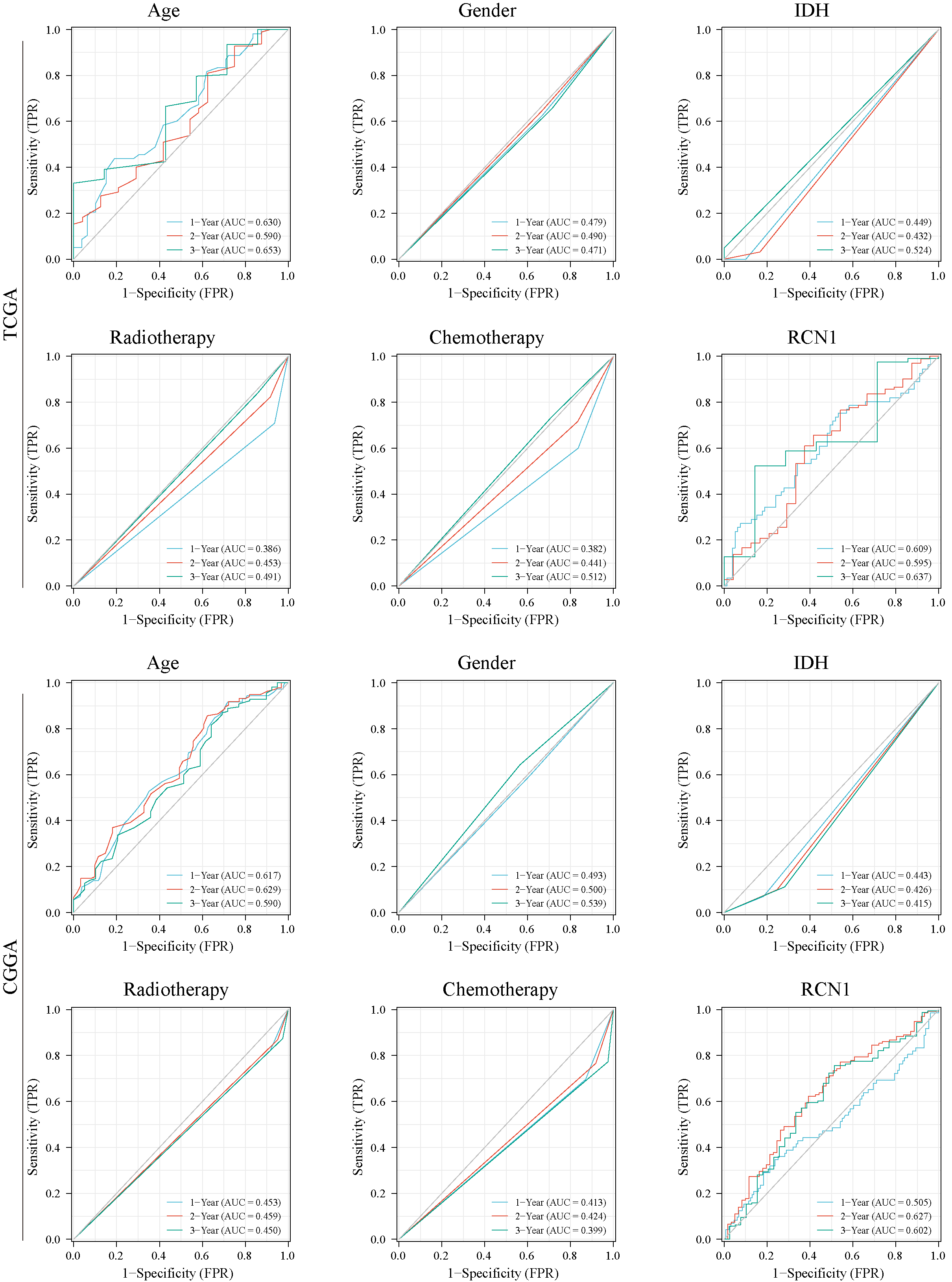

Supplement: Supplementary file 1 [file Image1.TIF]
